# To evaluate efficacy and safety of amphotericin B in two different doses in the treatment of post kala-azar dermal leishmaniasis (PKDL)

**DOI:** 10.1371/journal.pone.0174497

**Published:** 2017-03-29

**Authors:** Vidya Nand Rabi Das, Niyamat Ali Siddiqui, Biplab Pal, Chandra Shekhar Lal, Neena Verma, Ashish Kumar, Rakesh Bihari Verma, Dhirendra Kumar, Pradeep Das, Krishna Pandey

**Affiliations:** 1 Department of Clinical Medicine, Rajendra Memorial Research Institute of Medical Sciences (Indian Council of Medical Research), Agamkuan, Patna, Bihar, India; 2 Department of Biostatistics, Rajendra Memorial Research Institute of Medical Sciences (Indian Council of Medical Research), Agamkuan, Patna, Bihar, India; 3 Department of Pharmacy Practice, National Institute of Pharmaceutical Education and Research, Hajipur, Bihar, India; 4 Department of Biochemistry, Rajendra Memorial Research Institute of Medical Sciences (Indian Council of Medical Research), Agamkuan, Patna, Bihar, India; 5 Department of Pathology, Rajendra Memorial Research Institute of Medical Sciences (Indian Council of Medical Research), Agamkuan, Patna, Bihar, India; 6 Department of Molecular Biology, Rajendra Memorial Research Institute of Medical Sciences (Indian Council of Medical Research), Agamkuan, Patna, Bihar, India; George Washington University School of Medicine and Health Sciences, UNITED STATES

## Abstract

**Background:**

Post kala-azar dermal leishmaniasis (PKDL) is a skin disorder that usually occurs among patients with a past history of visceral leishmaniasis (VL). Cases are also reported without a history of VL. There is no satisfactory treatment regimen available at present. We aimed to compare the efficacy and safety of amphotericin B in two different doses (0.5mg/kg vs 1mg/kg) in a prospective randomized trial in 50 PKDL patients.

**Methods:**

In this open label study 50 patients with PKDL, aged between 5–60 years were randomized in two groups. Group A received amphotericin B in the dose of 0.5 mg/kg in 5% dextrose, daily for 20 infusions for 3 courses at an interval of 15 days between each course and Group B received amphotericin B in the dose of 1mg/kg in 5% dextrose on alternate days, 20 infusions for 3 courses an interval of 15 days between each course and followed up for one year.

**Results:**

A total of 50 patients were enrolled, 25 in each of group A and group B. Two patients lost to follow up and three patients withdrew consent due to adverse events. The initial cure rate was 92% in group A and 88% in group B by intention to treat analysis and final cure rate by per protocol analysis was 95.65% and 95.45% in group A and group B respectively. Two patients each from either group relapsed. Nephrotoxicity was the most common adverse event occurring in both the groups.

**Conclusion:**

The lower dose appears to have fewer adverse events however, nephrotoxicity remains a problem in both regimens. The 0.5mg/kg regimen may be considered instead of the higher dosage however safer treatments remain critical for PKDL treatment.

## Introduction

Post kala-azar dermal leishmaniasis (PKDL) is a dermatological complication caused by the protozoal parasite genus Leishmania. The vector responsible for the development of visceral leishmaniasis (VL) and subsequently PKDL is a female Sandfly *Phlebotomus argentipes*. The disease is characterized by macular, papular and nodular lesions or a mixture of these all over the body, but face is the most common site of occurrence. PKDL can lead to severe disfigurement especially the face. Among all the lesions, nodular forms are probably a prominent source of disease transmission in Sudan and the Indian subcontinent [1]. Apart from skin rashes PKDL patients are healthy and do not have any physical limitation [2]. Previously it used to develop among patients with a past history of VL treated with sodium antimony gluconate (SAG) [3]. It also occurs with all the currently available therapies such as miltefosine [4], paromomycin [5], amBisome [6] and even in combination of miltefosine and paromomycin (unpublished). PKDL is considered as a reservoir of the parasite and plays a key role in the transmission of VL [7] thus, identification and prompt treatment of PKDL cases are required to interrupt the transmission, which in turn will be helpful to eliminate kala-azar from the Indian subcontinent.

Miltefosine is currently first line therapeutic regimen for the treatment of PKDL in India. It is the only available drug administered orally at a dose 2.5mg/kg/day (in children) or 100mg/day (adults) for 12 weeks. Gastrointestinal side effects and poor compliance are major limiting factors against its usage and it is contraindicated in pregnant and lactating women because of the risk of teratogenicity [8]. As PKDL occurs among the poorest of poor people so cost effectiveness is a major barrier to the widespread use of all the available regimens.

Amphotericin B at a dose of 1mg/kg for 60 to 80 infusions is another treatment option and is associated with adverse events such as nausea, vomiting, fever, rigor and other toxicities [9]. These complications carry the risk of noncompliance and treatment abandonment. Therefore, it could be hypothesized that these cases might work as a reservoir for parasites and play a role in the disease transmission. An incomplete treatment recipient may also promote drug resistance which is a growing crisis with all the antileishmanial drugs. All these consequences will ultimately hamper the ongoing effort of kala-azar elimination from the Indian subcontinent, which aims to eliminate kala-azar by 2017 [10]. With the view of the above, we therefore, tried to evaluate whether amphotericin B at a dose of 0.5 mg/kg daily for 20 infusions for 3 courses has efficacy and safety similar to that of amphotericin B administered at a dose of 1 mg/kg alternate day for 20 infusions for 3 courses. It is hypothesized that lowering the dose will produce lesser side effects [11]. The outcome of this study may provide sufficient evidence to the clinician and program makers to reduce the dose of amphotericin B for the treatment of PKDL. It may also be helpful to encourage treatment adherence and reduce the economic burden of the afflicted individual.

## Materials and methods

### Study design, sampling technique and sample size

This was an open label, non-comparative randomized, parallel arm study. Due to the limited numbers of cases of PKDL that are seen at the facility, the study could not be powered for non-inferiority. Safety analysis was based on the protocol-specified definition of the intent-to treat population, which included all patients who enrolled in the study and receiving the regimen. The actual sample size calculation was not attempted due to low incidence of PKDL and limited availability of resources. Moreover, therapeutic options for PKDL are very few and vital for the kalaazar elimination program. Therefore, the sample size was set 50 (25 in each arm).

Male and female patients aged between 5–65 years were included in the study. Baseline evaluation included complete haemogram, liver function tests, chest X-ray, serum urea and creatinine, serum sodium and potassium. Patients with any malignant disorder, seropositive for human immunodeficiency virus, hepatitis B and C, tuberculosis, and suffering from any other infectious diseases were excluded from the study. Pregnant or lactating women were also excluded. Randomization was done by using sequentially numbered sealed envelopes prepared from a computer-generated randomization sequence. Initial diagnosis of PKDL was based on the clinical examination and positive immuno-chromatographic rK39 strip test. The diagnosis was confirmed by visualization of Leishman Donovan bodies through a slit skin smear after giemsa staining under the microscope and measuring the parasitic burden by using quantitative real-time PCR (Q-PCR) [12]. Only qPCR positive cases were included in the study.

**Fig 1:**
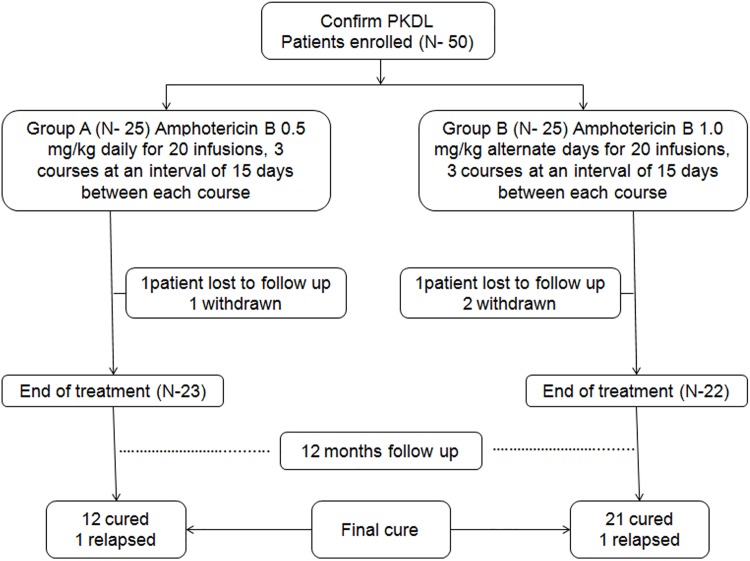
Patients flow diagram

A total 64 eligible patients were screened and 9 patients declined to participate and 5 patients did not meet the eligibility criteria due to the absence of parasite in skin smear. Therefore, we enrolled 50 patients in the study in two groups, 25 in group A and 25 in group B (Fig 1). Patients were assigned to receive 20 intravenous infusions of amphotericin B for 3 courses at an interval of 15 days between each course either at a dose of 0.5mg/kg daily (Group A) or 1mg/kg alternate days (Group B). They were admitted to hospital for the entire duration of each course. After completion of each course they were advised to return after 15 days. This was repeated in each subsequent course. All the patients were followed up at 3, 6, 9 and 12 months after the end of treatment.

### Study site

The study was conducted at the Rajendra Memorial Research Institute of Medical Sciences, Indian council of Medical Research (ICMR), Patna, India between 05.05.2014 to 04.02.16. The Ethics Committee of Rajendra Memorial Research Institute of Medical Sciences (RMRIMS), Patna approved the protocol. This study was conducted in compliance with the protocol. Informed consent form was administered in local language (Hindi). Written informed consent was taken from all the participants. In case of subjects <18 years of age written consent were taken from legal guardians. The individuals shown in this manuscript have given written informed consent (as outlined in PLOS consent form) to publish their pictures.

### Safety assessment

All the subjects were monitored daily for the incidence of adverse events. A biochemical and hematological investigation, including complete blood cell count, liver function test, serum creatinine, blood urea, blood sugar and electrolyte levels were done after 10 and 20 infusions of each course and at 3, 6, 9 and 12 months follow up. All the adverse events were graded according to the National Cancer Institute Common Terminology Criteria (NCI-CTC) AE (Adverse event), version three [13].

### Efficacy

Assessment of efficacy was done of by the initial cure as complete regression of papular and nodular lesions and/or no appearance of new macular lesions, together with a grade 0 parasite score in the dermal lesion and/or insignificant parasite burden by qPCR at the end of treatment. Parasitic load in tissue biopsy was also measured by qPCR before treatment and at the end of treatment in both the groups. Final cure constituted of disappearance of clinical signs and symptoms for PKDL, total re-pigmentation of macules and Grade 0 parasite score in dermal lesions at twelve months follow up. However, parasitic load was not estimated at follow up visit. Treatment failure was assumed when there was no improvement of the skin lesions at the end of 3 courses of treatment, including positive parasite in skin smear and/ or qPCR. Relapse was defined as the appearance of new lesions or reappearance of previously healed lesions within 12 months along with positive skin smear.

### Rescue therapy

Patients who were discontinued due to adverse events or relapsed at or before one year follow up received rescue treatment with miltefosine at the dose of 2.5mg/kg/day or 100mg/day for 12 weeks.

### Statistical analysis

Data was analyzed by using SPSS software (version 16). Descriptive statistics were used to describe continuous variables under the study. Intention-to-treat (ITT) and per-protocol (PP) analyses was used for assessment of initial and final cure with 95% confidence interval. Fisher’s exact test was used for categorical variables. Paired t tests were performed to compare the mean difference for continuous data before and after the treatment and Unpaired t test to compare mean for continuous variable between two groups. P value <0.05 was considered as statistical significance. The study was registered after enrollment of patients because we were not aware of the need of registration prior to recruitment. The authors confirm that all ongoing and related trials for this drug/intervention are registered.

This study was registered with the Clinical Trials Registry- India (CTRI), number CTRI/2016/05/006958.

## Results

Patients comprised of 24 males and 26 females. Median age of group A and B were 19 [Inter quartile range (IQR), 6.5] years and 20 (IQR, 12) years respectively. Forty patients had a past history of VL and ten patients did not report a history of having been treated for VL. Thirty patients had been treated with SAG, seven with miltefosine and three each with paromomycin, AmBisome, amphotericin B, for their past illness of VL. Nineteen patients had hypopigmented macular lesions, seven had nodular, six had papular and 18 had mixture of macular and papular lesions. The details of clinicoepidemilogical profiles of both the groups are depicted in Table 1.

**Table 1:** Demographics and clinical characteristics of PKDL patients (N = 50).

Variables	Group A (N = 25)	Group B (N = 25)	p value
Age in years			
Mean(±SD)	19.8(5.9)	22.25(9.5)	0.28
Sex			
Male	11(44%)	13(52%)	0.38
Female	14(56%)	12(48%)	
Past history of VL			
Yes	21(84%)	19(76%)	0.36
No	4(16%)	6(24%)	
Past history of VL			
Yes	13(52%)	8(32%)	0.12
No	12(48%)	17(68%)	
Previous treatment for VL			
SAG	13(62%)	17(89.5%)	0.19
Miltefosine	6(29%)	1(5.2%)	0.04
Paromomycin	0	1(5.2%)	-
Ambisome	1(4.7%)	0	-
Amphotericin B	1(4.7%)	0	-
Type of Lesion			
Macular	10(40%)	9(36%)	0.5
Papular	2(8%)	4(16%)	0.33
Maculopapular	8(32%)	10(40%)	0.38
Nodular	5(20%)	2(8%)	0.2

**VL:** Visceral leishmaniasis, **SAG:** Sodium antimony gluconate.

Parasitic load was found to be high in nodular lesions and low in macular lesions. The parasitic load have been significantly decreased after treatment (P<0.001). The details of parasitic load and threshold cycle (Ct) value are presented in Table 2.

**Table 2:** Parasitic load and Ct value of both the groups based on morphology.

	Group A (N = 25)			Group B (N = 25)		
Parasitic load/ μg of tissue	BT (Mean ± SD)	AT (Mean ± SD)	p value	BT (Mean ± SD)	AT (Mean ± SD)	p value
Macular	100372.5(12409.3)	9.0(4.6)	<0.001	96278(23300.0)	9.2(4.9)	<0.001
Papular	131392(5757.2)	11.5(0.2)	<0.001	116466.7(20869.2)	10.7(4.3)	<0.001
Nodular	162008.6(10675.6)	14.6(2.5)	<0.001	200005.5(20755.7)	13.1(5.3)	<0.001
Maculopapular	123276.8(18151.0)	12.4(1.6)	<0.001	117584.9(19453.5)	11.6(4.6)	<0.001
**Ct value**						
Macular	18.36(1.4)	37.6(1.3)	<0.001	18.2(2.0)	36.9(1.3)	<0.001
Papular	15.00(0.8)	35.2(0.5)	<0.001	15.2(1.0)	35.3(1.1)	<0.001
Nodular	13.00(0.4)	32.0(0.9)	<0.001	14.1(0.0)	33.0(0.2)	<0.001
Maculopapular	15.10(1.8)	34.9(1.1)	<0.001	15.1(2.4)	35.7(2.3)	<0.001

**BT:** Before treatment, **AT:** After treatment, **Ct:** Threshold cycle.

### Efficacy

Two patients, one from each group did not turn up after receiving first course. Another two patients withdrew consent due to frequent nausea and vomiting and one patient was withdrawn due to nephrotoxicity. On completion of first course, all the nodular and papular lesions were decreased in size almost equally in both the groups however, macular lesions remained unchanged. On completion of second course, most of the papular and nodular lesions were disappeared and half of the macular lesions were faded in both the groups and there was no appearance of new lesions. Apart from macular lesions of 10 patients (4 from group A and 6 from group B) all types of lesions were healed completely by the end of third course. The remaining macular lesions were disappeared at subsequent follow-up.

**Fig 2:**
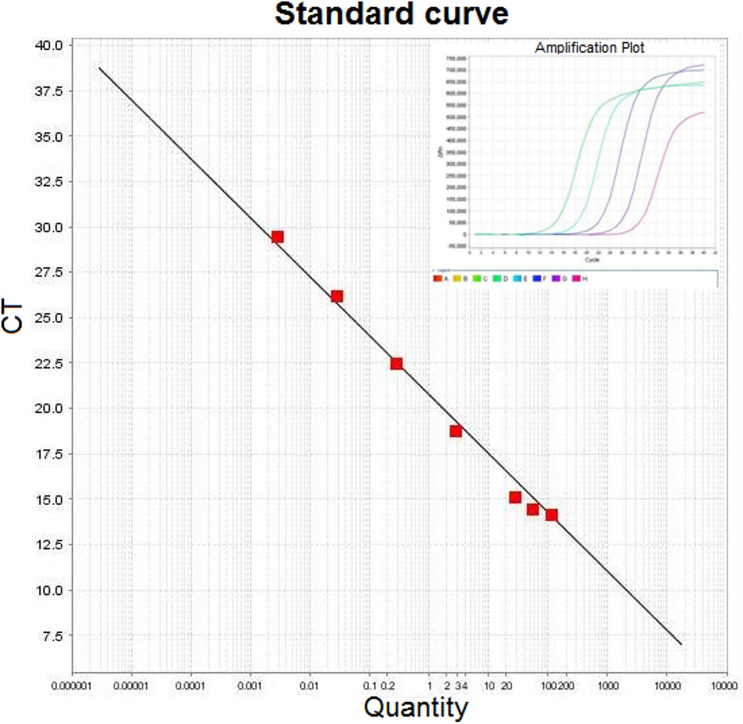
Parasite load (parasites/μg tissue DNA) in tissue biopsy samples were determined by quantitative real-time PCR (Q-PCR) using standard curve with SYBR Green. Extracted DNA from *L*. donovani were used for standard curve after 10-fold serial dilution. Ct- threshold cycle value; slope- 3.23; intercept- 25.08; R2- 0.991.

**Fig 3:**
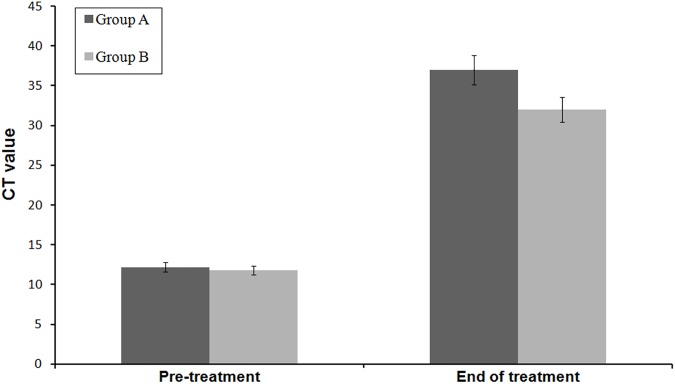
Parasitic load before treatment and at end of treatment shown by bar graph in which parasitic load is inversely proportional to Ct value.

**Fig 4:**
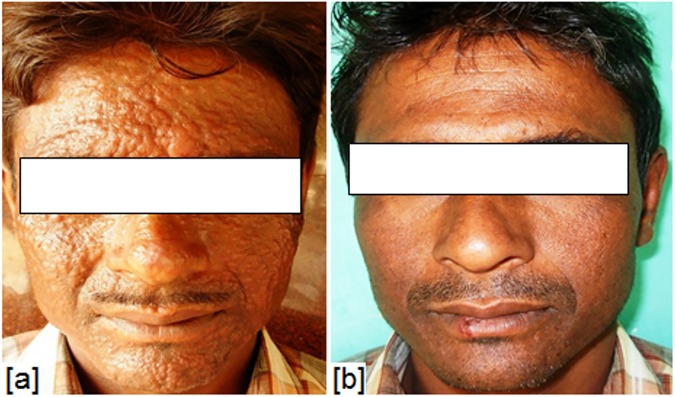
Skin lesions in a male patient- **(**a) before treatment, (b) after treatment with amphotericin B 0.5mg/kg/day.

**Fig 5:**
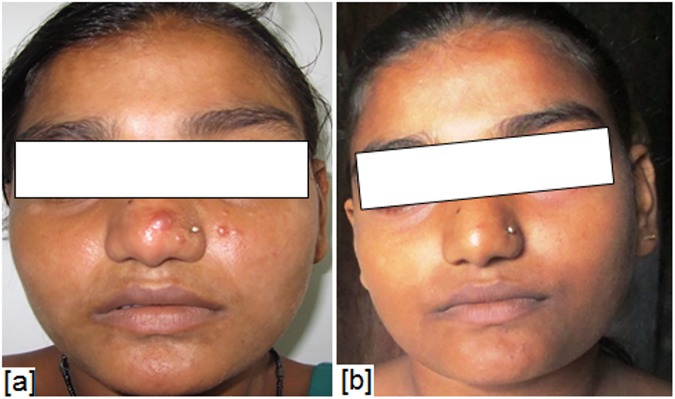
Skin lesions in a female patient- **(**a) before treatment, (b) after treatment with amphotericin B 0.5mg/kg/day.

Initial cure was achieved in 45 patients (23 in group A and 22 in group B) and cure rate by intention to treat analysis was 92% and 88% in group A and group B respectively. The parasite load in tissue biopsy showed that 8 parasites per gram of tissue (parasites/g) DNA in group A while 10 parasites/g of tissue DNA in group B (Figs 2 and 3). The final cure rate by per protocol analysis was 95.65% (95% confidence interval 79–99%) in group A (Figs 4 and 5) and 95.45% (95% confidence interval 78.2–99.2%) in group B. One patient each from group A and B relapsed at 9 months and 12 months follow up respectively. The summary of treatment response is presented in Table 3.

**Table 3:** Response to treatment with two different groups.

Response	Group A (N = 25)	Group B (N = 25)
Initial cure	92.0% (23/25)	88.0% (22/25)
Final cure	95.6% (22/23)	95.4% (21/22)
Relapse	4.3% (1/23)	4.5% (1/22)

### Safety

Nephrotoxicity was the predominant adverse events developed in both the groups. The baseline mean(±SD) serum creatinine was 0.77(0.18) mg/dl and 0.81(0.17) mg/dl in group A and Bespectively. The mean absolute value of serum creatinine was almost similar in both the groups at every cycle, however, the absolute mean value of group B was slightly higher in the second cycle as compared same cycle of group A. The frequency of occurrence of AEs in group A were higher in cycle 1 in compare to cycle 2 and 3 whereas, in group B the AEs were slightly higher in each cycle. Serious adverse events (SAEs) were observed in 52% (13/25) and 64% (16/25) patients in group A and group B respectively. All the SAEs were related to prolonged hospitalization due to elevated serum creatinine level. Number of episodes of fever and or rigor in a range of (1–2 times and >6 times) occurred during infusion was higher in group B as compared to group A. Other adverse events commonly occurring in both the groups were relative tachycardia, decrease serum potassium level, raise serum urea level, anorexia, eosinophilia, anemia, etc. The details of occurrence of adverse events are presented in Table 4.

**Table 4:** Frequency/ Percentage of all Adverse events in Group A and Group B.

Adverse events (AE)	Group A (N = 25)								Group A (N = 25)							
	Cycle 1 (n = 71)		Cycle 2 (n = 63)		Cycle 3 (n = 61)		FU	CF(%)	Cycle 1 (n = 84)		Cycle 2 (n = 68)		Cycle 3 (n = 90)		FU	CF(%)
	I	II	I	II	I	II			I	II	I	II	I	II		
Increased serum creatinine (mg/dl)																
1.2–1.5	1	1	1	1	1	1	0	6(2.9)	1	1	2	1	2	3	0	10(4.0)
1.5–2.0	4	5	2	6	2	3	0	22(10.8)	6	2	4	3	6	3	0	24(9.7)
>2.0	2	3	3	2	1	4	0	15(7.4)	3	5	2	3	4	4	0	21(8.5)
Decreased serum potassium (mmol/liter)																
<3.0	-	-	-	-	-	-	-	0	-	-	-	-	-	-	-	0
3.0–3.5	5	2	2	4	0	1	0	14(6.9)	3	4	3	2	5	4	0	21(8.5)
Fever and/or rigor																
1–2 times	2	1	2	1	2	0	0	8(3.9)	1	4	2	1	2	2	0	12(4.8)
3–6 times	3	5	3	2	4	5	0	22(10.8)	2	5	2	3	4	4	0	20(8.1)
>6 times	2	3	1	3	4	3	0	16(7.9)	6	3	3	4	2	5	0	23(9.3)
Nausea and vomiting																
1–2 times	1	2	2	1	1	1	0	8(3.9)	1	0	1	0	2	1	0	5(2.0)
3–6 times	2	0	1	0	1	0	0	4(1.9)	2	3	1	3	2	4	0	15(6.0)
>6 times	0	0	0	0	0	1	0	1(0.4)	1	2	1	1	0	2	0	7(2.8)
Elevation of hepatic enzymes (ALT/AST)	5	3	3	3	5	4	1	24(11.8)	3	2	2	2	4	3	0	16(6.5)
Relative tachycardia	3	2	3	3	2	3	0	16(7.9)	4	3	3	1	4	2	0	17(6.9)
Hyperuremia	3	2	2	1	1	2	0	11(5.4)	1	3	2	2	3	3	0	14(5.6)
Eosinophilia	1	0	1	2	1	1	2	8(3.9)	0	1	2	1	2	0	2	8(3.2)
Abdominal pain	0	1	1	0	1	1	0	4(1.9)	1	0	1	0	1	0	0	3(1.2)
Anorexia	1	0	1	1	0	1	0	4(1.9)	1	1	0	2	1	0	0	5(2.0)
Anemia	0	1	0	0	1	0	1	3(1.4)	1	0	1	0	0	1	0	3(1.2)
Gastritis	0	0	0	1	0	0	0	1(0.4)	1	2	1	2	1	2	0	9(3.6)
Headache	1	0	0	0	0	0	0	1(0.4)	1	1	0	1	0	1	0	4(1.6)
Others	3	1	2	2	2	1	3	14(6.9)	1	2	2	1	1	0	2	9(3.6)
Overall	39	32	30	33	29	32	7	202(100)	40	44	35	33	46	44	4	246(100)

**N:** Total no. of patients, **n:** Frequency of occurrence of AEs, **FU:** Follow up, **CF:** Cumulative frequency, **I:** Day 10 of each cycle, **II:** Day 20 of each cycle, **AST:** Aspartate transaminase, **ALT:** Alanine transaminase.

## Discussion

Amphotericin B is a polyene antibiotic, having antimycotic and antileishmanial action, first isolated in 1955 from Streptomyces nodosus [14]. It is reported that amphotericin B is a membrane-active drug that forms channel-like structures (pores) spanning the lipid bilayer thereby increasing the permeability of cell membranes, causing leakage of ions and small solute molecules followed by cell death [15]. The major drawback associated with its treatment is metabolic abnormality such as nephrotoxicity and hypokalemia. Sometimes nephrotoxicity occurs which can result in acute renal injury [16]. The remarkable advances in research led to the formulation of liposomal amphotericin B (AmBisome). Due to its excellent safety and efficacy profile WHO has recommended the use of liposomal amphotericin B as a first line therapy in the treatment of visceral leishmaniasis in the Indian subcontinent [17]. National Vector Borne Disease Control Program (NVBDCP), India recommends amphotericin B at 1mg/kg (60–80 doses) as a second line therapy after miltefosine for the treatment of PKDL. In patients whom miltefosine is contraindicated amphotericin B is the treatment of choice. In the present study, we tried amphotericin B at 0.5mg/kg for 60 doses which is a cumulative dose of 30mg/kg for group A, whereas 1mg/kg for 60 doses with a cumulative dose of 60mg/kg for group B.

Amphotericin B in VL either daily or alternate days at 1mg/kg has shown consistently high efficacy with a cure rate of >95% [18, 19]. However, the alternate day regimen is considerably preferable in Indian Sub-continent due to concern about its tolerability [20]. An earlier study of amphotericin B at 0.5mg/kg/day has been found to be superior to antimony in the treatment of VL with an initial cure rate of about 100% [21]. Further escalating the dose to 0.75mg/kg for the treatment of VL, showed equally efficacious to 1mg/kg moreover, it is proved to be safe, shorter duration and also reduces cost of treatment [22]. Furthermore, Amphotericin B at a dose of 1mg/kg has also shown excellent safety and efficacy for the treatment of Indian PKDL. Eleven patients were treated with amphotericin B at1mg/kg and final cure rate was 100% (11 of 11 patients were cured). All the side effects were mild in intensity and proved superior to SAG [9]. In Sudan Liposomal amphotericin B at 2.5mg/kg daily for 20 courses showed promising result in the treatment of PKDL with 83% final cure rate without any AEs [23]. The efficacy obtained in our study is concordant with the study conducted by Sundar et.al where miltefosine was tried at100mg/kg for 12 weeks in Indian PKDL patients; with 93% cure rate along with treatment failure in one case [24]. Another study conducted by Ramesh et.al administered miltefosine in 26 PKDL patients, 23 was cured and one reported treatment failure [25].

Acute nephrotoxicity developed in both the groups. None of the patients experienced renal failure. Nephrotoxicity led to withholding the treatment till the serum creatinine became normal, thereby increasing the treatment duration which poses economic burden to the poor people. Infusion related reactions such as fever and or rigors, nausea and vomiting, which are expected with any amphotericin B containing formulation, were more frequent in group B compared to group A. All the AEs and SAEs were managed without referral to another hospital. Patients need to be admitted for a minimum of 60 days to complete the treatment, duration may further enhance due to adverse events mainly nephrotoxicity associated with the treatment. Which will increase the work loss days of daily wage earners thereby poses a high economic burden to the poor people affected with PKDL. These are the major drawback with amphotericin B treatment.

The Q-PCR data indicates that group A patients having a higher parasitic clearance in compared to group B. rK39 is a reliable, noninvasive and field based test for screening of VL and PKDL. The major drawback is, it becomes positive in treated cases of VL. As regards to noninvasive samples, urine can be used in place of blood and serum in rK39 based strip test for diagnosis of VL, but it has not been tested for PKDL [26]. Slit aspirate is another simple and minimally invasive method showing high sensitivity and specificity with rK39 strip test, however microscopical observation of slit aspirate sample offers low sensitivity (60%) [12]. The sensitivity and specificity of molecular diagnostic methods such as PCR/qPCR are very high [27,28]. These molecular tests give equally reliable results using tissue sample or skin slit aspirates however, high initial investment and high cost per test are the major limiting factors.

Even when the country is under elimination mode, there are challenges in PKDL case detection and management, and still number of interventions needs to be implemented under the existing national program to achieve the elimination target by 2017. Early detection of PKDL with the help of trained dermatologist/clinicians/pathologist and possible linkages existing under health system network need to be explored and utilized on priority to address this gap. Recently a study by Ramesh et. al reported that the efficacy of miltefosine is decreasing substantially in the treatment of PKDL with a cure rate of 85% [29]. A Similar trend of decrement had been found in the treatment of VL with miltefosine, the final cure rate dropped from 94% (phase III trial during 1999–2000) to about 90% [30]. Therefore, a better and safer treatment regimens need to be developed for the treatment of PKDL in the Indian sub-continent.

The major limitation of this study was the sample size. The other limitation was qPCR, which was performed for only rK39 and microscopy positive subjects. QPCR was not performed at the time of final follow up.

In conclusion, the results suggest that amphotericin B at 0.5mg/kg daily regimen is highly effective, shortened duration of hospitalization and also able to minimize some of the drawbacks related to its tolerability. However, increased serum creatinine remains a common problem with both the regimens. Hence, monitoring of serum creatinine at least once in the midway of each course of therapy is recommended or based on treating clinicians depending on the clinical signs and symptoms of the patients. Therefore, amphotericin B at 0.5mg/kg regimen may be considered as one of the options for the management of PKDL where miltefosine is contraindicated. Further, multi center randomized trial with the appropriate sample size need to be conducted to precisely confirm the dose.

## Supporting information

S1 Checklist Consort 2010 checklist. (DOC)

S1 Protocol Study protocol. (DOCX)
