# Supplementary material for: To evaluate efficacy and safety of amphotericin B in two different doses in the treatment of post kala-azar dermal leishmaniasis (PKDL)
Source: PLoS One. 2017 Mar 29;12(3):e0174497. doi: 10.1371/journal.pone.0174497 (PMC5371363; doi:10.1371/journal.pone.0174497)
Supplement: S1 Protocol — (DOCX) [file pone.0174497.s002.docx]

**PROJECT TITLE:** To evaluate efficacy and **s**afety of Amphotericin B in two different doses in the treatment of Post Kala-Azar Dermal Leishmaniasis (PKDL)

PI- Dr V N R Das

Co-Investigator- K Pandey, N Verma, C S Lal, P K Sinha, & P Das.

- ***Primary Objective:***
- To evaluate the efficacy and safety of Amphotericin B in different doses in the treatment of PKDL
- **Secondary Objectives:**
- To assess the efficacy of two doses in the treatment of PKDL.
- To evaluate parasite load in the skin lesion by qPCR.
- To evaluate the safety of drug with the help of clinical, haematological and biochemical parameters during the course of treatment.

**Background**

Post Kala-azar Dermal Leishmaniasis is a late complication of Kala-azar after treatment.^1^ It may also develop without history of Kala-azar directly from asymptomatic cases of Kala-azar.^2^ PKDL manifests as depigmented macules which may start as dots and then develop into patches or papules and nodules.^3^ The lesions are found on face, hand, back, thigh, and mucous membrane of tongue and rarely on genitalias.^4^ Cases of PKDL are of considerable epidemiological importance, and acting as reservoirs for transmission of Leishmania parasite.^5^ Treatment of PKDL is very difficult with existing anti-leishmanial drugs.^6^ SAG and Miltefosine is highly toxic in the treatment of PKDL, required prolong treatment and also shown relapse after along gap of treatment.^7^ Amphotericin B is superior than SAG and cure rate high without relapse.^8^

In the Indian subcontinent, immunological factors predisposing patients with VL to PKDL remain poorly characterized, with the disease pathology often being attributed to parasite-specific cell-mediated (CMI) responses (Ramesh et al., 2007). Sudanese patients with PKDL respond Leishmania antigen (Ismail et al., 1983). Whereas studies on immune responses in Indian PKDL are not so clearly defined and even contradictory (Halder et al., 1983). Clinical reports have not identified sex, age or racial origin as defining factors in PKDL. However, genetic disposition might be involved as indicated in Sudan where studies identified linkage of the IFN- γ receptor to PKDL, but not to VL (Mohamed et al., 2003). The immunological status of PKDL patients is complex with mixed T cell responses and elevated levels of IL-10 and TNF-α (Ansari et al, 2006; Ganguly et al, 2008).

In this study, we can propose treatment of PKDL with Amphotericin B in two different doses as 0.5 mg/kg body wt daily compared with 1mg/kg body wt on alternate day.

**Study Design**

The study is a randomized, open label study, designed to establish that Amphotericin B therapy can be drug of choice for the treatment of PKDL cases. In this study to assess safety and efficacy of Amphotericin B for the treatment of Post Kala-azar Dermal Leishmaniasis in two different doses modalities.

**Patients’ eligibility:** The patients will be selected from the out-patient department of Rajendra Memorial Research Institute of Medical Sciences (ICMR), Patna, India. They will be admitted in the ward and treated according to the group given (A and B).

*Inclusion criteria:*

- Both sex patients aged 5-60 years
- Parasitologically confirmed Post kala azar dermal leishmaniasis (PKDL),
- All types of PKDL.

*Exclusion criteria:*

- Pregnant and lactating females.
- Patients not willing to participate.
- Individuals who were sero positive for HIV
- Individuals with a serious concurrent infection (e.g., tuberculosis or bacterial pneumonia) were excluded from the study.
- Thrombocyte count <100 x 10^9^/l
- Leukocyte count <2.5 x 10^9^/l
- Hemoglobin < 6.0 g/100 ml
- ASAT, ALAT, AP >3 times upper limit of normal range
- Bilirubin >2 times upper limit of normal range
- HbsAg, HCV and HIV positive
- Serum creatinine or BUN >1.5 times upper limit of normal range
- Relapsed PKDL cases

**Sample Size**: 50 in numbers after reviewing inclusion and exclusion criteria, eligible patients will be enrolled in two different groups (25 in each group).

**Test Arm: “**Group A” will receive Amphotericin B in the dose of 0.5 mg/ Kg body weight daily for 20 infusions at 15 days interval followed 3 courses.

**Control Arm***:* “Group B” will receive Amphotericin B in the dose of 1mg/kg body weight for 20 infusions in 5% dextrose on alternate days in 3 courses at 15 days interval.

**Randomization:**

All the patients will be administered an informed consent before starting any trial related activities. Open label un-blind randomization will be done through sequentially numbered sealed envelopes prepared from a computer-generated randomization sequence.

All routine investigations like TC/DC(WBC), haemoglobin, platelet count and biochemical investigations like serum bilirubin, SGPT, SGOT, BUN, serum creatinine, sodium and potassium will be done on 0, 15 , 30 days.

**Study assessments:**

***Safety:*** Safety will be assessed by collection of the following:

- All serious adverse events (SAEs) from time of admission through 12-month follow-up visit after end of treatment (EOT)
- All adverse events from time of first dose through EOT
- Clinical vital signs daily

***Efficacy:*** Efficacy will be assessed by collection of the following:

- **Parasitological cure**: defined as clearance of parasite from the dermal lesions that is Grade 0 parasite score in the dermal lesion at the end of treatment.
- **Final Clinical cure**: Define as total disappearance of skin lesion and Grade 0 parasite score at twelve months follow up.

**Laboratory investigational plan**

- Parasite Evaluation by Microscopy of lesion tissue: At Day 0 and EOT in both the groups and then every follow up visit till 12 months.
- Biochemical Evaluation: serum bilirubin, SGPT, SGOT, BUN, serum creatinine, sodium and potassium during Study period (Day 0, Day 15 , Day 30) of every course of treatment and then every follow up visit till 12 months in both the groups.
- Haematological Evaluation: Routine Haematological investigations like TC/DC (WBC), haemoglobin, platelet count during Study period (Day 0, Day 15, Day 30) of every course of treatment and then every follow up visit till 12 months in both the groups.
- Parasite load: By qPCR at day 0 and at the end of the treatment at lesion tissue .

**Withdrawal during study**: Any PKDL patient who develops the following will be withdrawn from the study:

- ASAT, ALAT, AP >3 times upper limit of normal range
- Bilirubin >2 times upper limit of normal range
- Serum creatinine or BUN >1.5 times upper limit of normal range
- Any serious adverse event
- Subject’s withdrawal if there willingness to discontinue from participation in study

**Expected Outcome:** Assessment of Amphotericin B (0.5 mg/kg body wt) low dose as a safe and effective for treatment of all types PKDL

**Duration of the study**: Three years

**Estimated Budget:** 10 Lakhs

**References**

1. [Ramesh V](http://www.ncbi.nlm.nih.gov/pubmed?term=Ramesh%20V%5BAuthor%5D&cauthor=true&cauthor_uid=21561437), [Katara GK](http://www.ncbi.nlm.nih.gov/pubmed?term=Katara%20GK%5BAuthor%5D&cauthor=true&cauthor_uid=21561437), [Verma S](http://www.ncbi.nlm.nih.gov/pubmed?term=Verma%20S%5BAuthor%5D&cauthor=true&cauthor_uid=21561437), [Salotra P](http://www.ncbi.nlm.nih.gov/pubmed?term=Salotra%20P%5BAuthor%5D&cauthor=true&cauthor_uid=21561437). Miltefosine as an effective choice in the treatment of post-kala-azar dermal leishmaniasis. [Br J Dermatol.](http://www.ncbi.nlm.nih.gov/pubmed/21561437) 2011 Aug; 165(2):411-4. doi: 10.1111/j.1365-2133.2011.10402.x.
2. Ramesh V, Mukherjee, A Post kala-azar dermal leishmaniasis (1995). Int J Dermatol; 34:85-91.
3. Zijlstra, EE and El-Hassan, AM (2001).Leishmaniasis in Sudan: Post kala-azar dermal leishmaniasis.Trans R Soc Trop Med Hyg; 95(Suppl 1):S59-76.
4. Zijlstra, EE, Musa AM, Khali EA, El-Hassan, IM (2003).Post kala-azar dermal leishmaniasis .Lancet; 3(2):87-98.
5. [Thakur CP](http://www.ncbi.nlm.nih.gov/pubmed?term=Thakur%20CP%5BAuthor%5D&cauthor=true&cauthor_uid=9425363), [Narain S](http://www.ncbi.nlm.nih.gov/pubmed?term=Narain%20S%5BAuthor%5D&cauthor=true&cauthor_uid=9425363), [Kumar N](http://www.ncbi.nlm.nih.gov/pubmed?term=Kumar%20N%5BAuthor%5D&cauthor=true&cauthor_uid=9425363), [Hassan SM](http://www.ncbi.nlm.nih.gov/pubmed?term=Hassan%20SM%5BAuthor%5D&cauthor=true&cauthor_uid=9425363), [Jha DK](http://www.ncbi.nlm.nih.gov/pubmed?term=Jha%20DK%5BAuthor%5D&cauthor=true&cauthor_uid=9425363), [Kumar A](http://www.ncbi.nlm.nih.gov/pubmed?term=Kumar%20A%5BAuthor%5D&cauthor=true&cauthor_uid=9425363). Amphotericin B is superior to sodium antimony gluconate in the treatment of Indian post-kala-azar dermal leishmaniasis. [Ann Trop Med Parasitol.](http://www.ncbi.nlm.nih.gov/pubmed/9425363) 1997 Sep; 91(6):611-6.
6. Olliaro, P.L. and W.R.Taylor (2003). Antimonial compounds from bench to bedside. J Exp Biol; 206:3753-3759.
7. Karin, S. and Croft, S.L. (2005).In vitro and in vivo interactions between miltefosine and other antileishmanial drugs. Antimicrob Agents Chemother; 50(1):73-79.
8. Ramesh V, Singh R, Salotra P (2007) Post Kala- azar dermal leishmaniasis – an appraisal. Top Med Int Health 12: 848- 51
9. Ismail A, El Hassan AM, Kemp K et al. (1999) Immunopathology of Post Kala- azar dermal leishmaniasis (PKDL): T- cell phenotypes and cytokine profile. J Pathol 189:615-22
10. Halder JP, Ghose S, Saha KC et al. (1983) Cell- mediated immune response in Indian Kala- azar and Post Kala- azar dermal leishmaniasis. Infect Immun 42: 702-7
11. Mohamed HS, Ibrahim ME, Miller EN et al. Genetic susceptibility to visceral leishmaniasis in the Sudan: linkage and association with IL4 and IFNGR 1. Genes Immun 2003; 4: 351-5
12. Ansari NA, Ramesh V, Salotra P. Interferon (IFN) - gamma, tumor necrosis factor- alpha, interleukin- 6, and IFN- gamma receptor 1 are the major immunological determinants associated with Post Kala- azar dermal leishmaniasis. J Infect Dis 2006; 194: 958- 65
13. Ganguly S, Das NK, Panja M et al. Increased levels of interleukin- 10 and IgG3 are hallmarks if Indian Post Kala- azar dermal leishmaniasis. J Infect Dis 2008; 197:1762-1771
